# The future of routine immunization in the developing world: challenges and opportunities

**DOI:** 10.9745/GHSP-D-14-00137

**Published:** 2014-12-02

**Authors:** Angela K Shen, Rebecca Fields, Mike McQuestion

**Affiliations:** a United States Agency for International Development and United States Department of Health and Human Services, Washington, DC, USA; b Maternal and Child Survival Program, Washington, DC, USA; c Sabin Vaccine Institute, Washington, DC, USA

## Abstract

Vaccine costs in the developing world have grown from < US$1/child in 2001 to about $21 for boys and $35 for girls in 2014, as more and costlier vaccines are being introduced into national immunization programs. To address these and other challenges, additional efforts are needed to strengthen 8 critical components of routine immunization: (1) policy, standards, and guidelines; (2) governance, organization, and management; (3) human resources; (4) vaccine, cold chain, and logistics management; (5) service delivery; (6) communication and community partnerships; (7) data generation and use; and (8) sustainable financing.

## BACKGROUND

Four decades ago in 1974, the World Health Organization (WHO) launched the Expanded Programme on Immunization (EPI). The EPI blueprint laid out the technical and managerial functions necessary to routinely vaccinate children with a limited number of vaccines, providing protection against diphtheria, tetanus, whooping cough, measles, polio, and tuberculosis, and to prevent maternal and neonatal tetanus by vaccinating women of childbearing age with tetanus toxoid. The purpose of EPI was simple and straightforward—to deliver multiple vaccines to all children through a simple schedule of child health visits.^1^ At the time, basic health systems in most lower- and lower-middle income countries (LLMICs) were weak to nonexistent. Vaccine coverage levels among children younger than 1 year of age were less than 5%.^2^ By 1990, most LLMICs had institutionalized immunization programs based on the EPI blueprint. In 1991, the global target of vaccinating 80% of the world's children was declared to have been met, saving millions of lives. The capacities and capabilities of countries built through the EPI blueprint were responsible for such significant gains.^2^

Since then, more vaccines have been added to national immunization schedules, and the contribution of immunization programs to ongoing declines in infant and child mortality has increased commensurately.^3^^,^^4^ As of 2014, WHO has recommended that all immunization programs add vaccines against hepatitis B, *Haemophilus influenzae* type b, *Streptococcus pneumoniae*, rotavirus, rubella, and human papillomavirus (in girls). However, the full benefits of these vaccines has yet to be realized. WHO estimates 29% of deaths among children 1–59 months of age are vaccine-preventable.^5^ This gap is due largely to systems weaknesses in immunization programs that can be improved. With the addition of new vaccines, the complexity and costs of routine immunization increase.^2^^,^^3^^,^^6^^,^^7^ More vaccinations to protect against more diseases requires additional and better communication between health workers and caregivers as well as greater cold chain capacity. New vaccines and doses that expand immunization beyond infancy extend the benefits of vaccines across the life spectrum into adolescence and adulthood. Moreover, health systems in general are becoming more complex as new programs and services are added.

29% of deaths among children 1–59 months old are vaccine-preventable.

Fulfilling the vision of EPI requires sustained investments in routine immunization.^8^ Since its inception, expanded immunization has been a continuous and progressive story building on the fundamental managerial and technical health systems building blocks of leadership and governance; financing; service delivery; health workforce; products, vaccines, and technologies; and information systems. Additionally, the economic benefits of immunization are significant. Healthier individuals are, in the long term, more productive contributors to a country's wealth, particularly as countries rapidly transition from high to lower mortality levels.^9^

Despite substantial success, there remain numerous challenges to the ultimate effectiveness of routine immunization. These fall into the following programmatic areas: (1) policy, standards, and guidelines; (2) governance, organization and management; (3) human resources; (4) vaccine, cold chain, and logistics management; (5) service delivery; (6) communication and community partnerships; (7) data generation and use; and (8) sustainable financing. This paper describes experiences and challenges in these areas. There is much to learn from decades of valuable experience in immunization that can potentially be applied to the “bigger picture” of primary health care. Investment in routine immunization has the potential to both optimize the programs that deliver the benefits of lifesaving vaccines and bolster the underlying health systems on which they rest.

## WHAT IS ROUTINE IMMUNIZATION?

Routine immunization is the foundation through which countries provide access to lifesaving vaccines and control and eradicate vaccine-preventable diseases.^2^^,^^3^^,^^6^^,^^8^ It is the process of timely vaccination on a regular basis with vaccines considered important for a given country to reduce morbidity and mortality. This process is enabled by a country's health system and maintained through a set of management subsystems needed to continuously supply the full complement of scheduled vaccines, monitor their safety, control population coverage, and measure their epidemiological impact. Essential components of immunization systems align with fundamental health systems components and function within the context of broader health and social welfare systems.

Routine immunization both depends upon and effectively strengthens the health systems through which they are delivered.

A strong routine immunization platform has the potential to benefit the overall health system by generating policy and financing innovations, robust logistic and cold chain systems, and skilled human resources, particularly in the areas of management, surveillance, and regulation. Routine immunization creates opportunities to educate parents about the benefits of vaccines and other health services. Engaging the community in planning, financing, and delivering the program can increase equity and build trust in the government's ability to deliver immunizations. Immunization trust can lead to generalized trust and rising expectations for other government services.^10^ The development challenge is to ensure the investments materialize, are sustained, and result in quality and reliable immunization programs that help drive the broader socioeconomic development of countries.

The continued success of routine immunization depends on the availability of vaccines as well as the human, financial, and material resources needed to effectively deliver and use them. To achieve and maintain a country's immunization objectives, adequate resources must be forthcoming every year for an ever-expanding birth cohort. Ideally, those resources are obtained in sustainable fashion. Dependence on support from external partners, with their shifting institutional priorities, results in precarious management and uneven performance.^11^ An enabling environment, even in the poorest countries, depends on the political will of decision-makers. Paradoxically, political will declines if funding allocations are based strictly on morbidity and mortality burdens. This may put a high-performing immunization program at a disadvantage for securing the funding it needs to sustain high performance.^12^

## CHALLENGES TO ROUTINE IMMUNIZATION AND LESSONS LEARNED

The programmatic goal of routine immunization is to ensure that vaccination services are accessible, available, acceptable, and affordable to users in an efficient and effective manner. At a time when health systems weaknesses are highlighted by outbreaks of measles^13^ and, more recently, Ebola virus disease, current platforms including routine immunization investments provide potential to help strengthen systems sustainability.^14^

**Figure 1.**
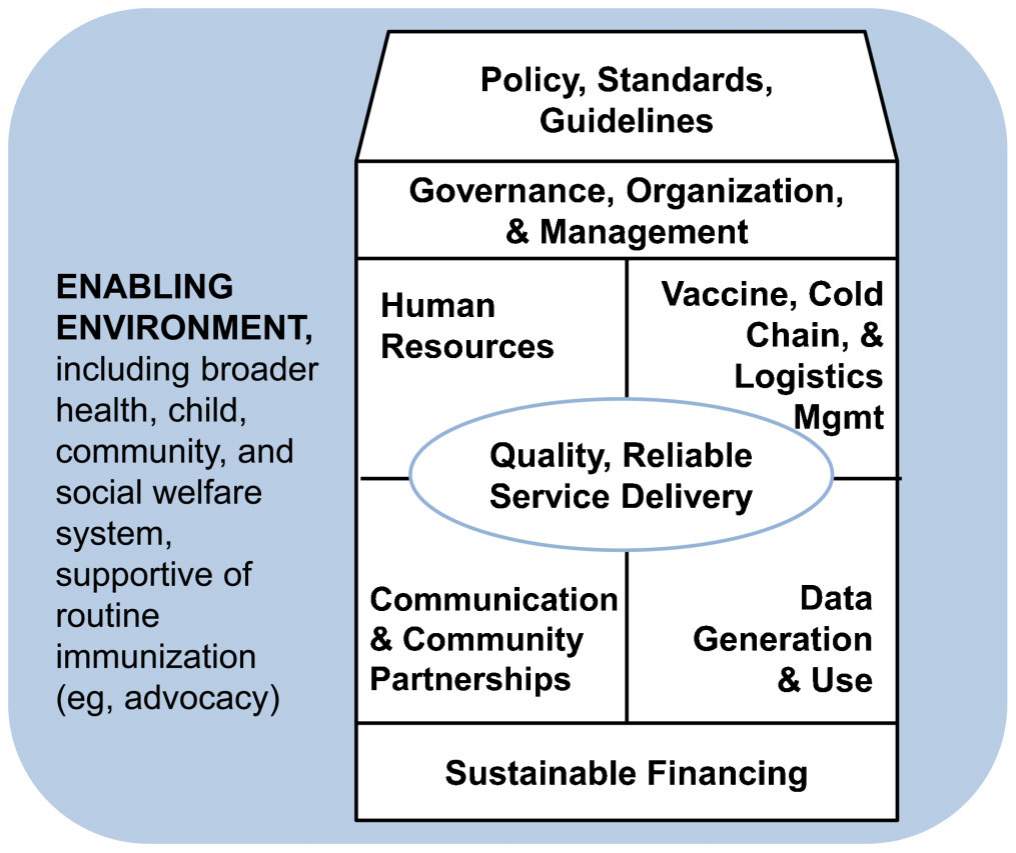
Critical Elements of Routine Immunization Programs

Despite the maturity of EPI, immunization systems as part of broader health systems have become fragile as they face new challenges and struggle to balance country needs with the achievement of global coverage goals and accelerated disease control efforts (eg, polio eradication, measles elimination). We describe some of these prominent challenges and successful approaches in the context of 8 core components of routine immunization (Figure 1):

- Policy, standards, and guidelines
- Governance, organization, and management
- Human resources
- Vaccine, cold chain, and logistics management
- Service delivery
- Communication and community partnerships
- Data generation and use
- Sustainable financing

### Establishing Policies, Developing Standards, and Providing Guidelines

From the advent of EPI, WHO has played a critical supporting role to countries, particularly LLMICs. WHO generates global-level policies, standards, and technical guidance for immunization and actively helps countries (member states) introduce and adapt them as appropriate. WHO has no direct policy-setting authority at the country level; each country is expected to establish its own immunization policies, standards, and guidelines.

National policymaking capacity varies considerably across LLMICs. Concerted efforts have been made in recent years to establish and formalize National Immunization Technical Advisory Groups (NITAGs). Comprised of top biomedical, academic, and clinical professionals, the primary function of NITAGs is to guide the development of national immunization policies, guidelines, and standards and to inform program decision-making, including recommendations on vaccine introduction and immunization schedules. Moreover, their recommendations should be evidence-based and generated through transparent processes.^15^ Such advisory bodies have long existed in industrialized countries but are new to LLMICs. As of 2012, 38 of 102 LLMICs reported to WHO that they had NITAGs that met 6 process criteria: legislative or administrative basis for the advisory group, formal terms of reference, diverse expertise/representation (eg, pediatrics, public health, epidemiology) among core members, number of meetings per year, circulation of the agenda and background documents at least 1 week prior to the meeting, and mandatory disclosure of any conflict of interest.^16^ Progress toward these process indicators strengthen NITAGS, an important tool in strong national immunization programs.

In the absence of a NITAG, de facto policies sometimes emerge in the form of recommended practices and procedures that appear in training materials, supervisory checklists, and data management and reporting tools.

NITAGs are distinct from Interagency Coordinating Committees (ICCs), which are mechanisms to coordinate immunization partner inputs. NITAGs are both a technical resource and deliberative body. They support national authorities and decision-makers to make evidence-based decisions, for example, on whether to introduce a new vaccine. As NITAGs evolve, they mirror immunization program development. Their advice helps managers set priorities and strategies tailored to epidemiological needs and programmatic capacities.

Regulation and oversight is another area central to well-functioning routine immunization platforms. Many LLMICs rely on the WHO vaccine prequalification process and UNICEF procurement mechanisms to ensure purchase of safe vaccines. A growing number of countries are increasing their investments in national regulatory authorities (NRAs) and associated regulatory systems (eg, laboratories). Strong NRAs are necessary if countries are to self-procure and ensure a reliable supply of quality vaccines.^17^

### Building Strong Governance, Organization, and Management

National governments typically have the responsibility for leading and overseeing routine immunization. However, immunization is best seen as a shared responsibility of individuals, communities, and governments. Many other partners may be involved. For example, the public sector is often responsible for purchasing routinely recommended vaccines and providing these to both public and private-sector providers (nonprofit and for-profit).^18^ Maintaining active representation from all partners and keeping the focus on routine immunization has been a continuing challenge. External partners tend to be most concerned with accomplishing specific, time-limited events, such as mass campaigns, new vaccine launches, or training events. In the most dependent LLMICs, financing and technical support for routine immunization is closely geared to these externally mandated outputs.

In most countries, the national immunization program provides managerial and technical leadership and serves a wide array of functions, including developing standards and guidelines; securing vaccine supply and distribution; preparing training materials and supervisory tools and implementing training; developing and carrying out communication strategies; planning the introduction of new vaccines; organizing immunization campaigns; collecting, analyzing, and providing reports on vaccine coverage, disease surveillance, and budget execution; and maintaining relationships with external agency counterparts. In some countries, these functions are fragmented, split among different units or divisions of the health ministry or even across multiple ministries. This affects the efficiency and effectiveness of program functions and linkages.

Strong managerial and technical leadership is required to manage the many partners and functions involved in national immunization programs.

Increasingly, health systems are being decentralized. Subnational health teams, often at the district level, are responsible for managing and providing the fully array of basic health services, including immunization. In principle, decentralization makes routine immunization more robust by bringing the management of services closer to the populations being served. In practice, immunization and other decentralized public services often suffer from a dearth of capable managers.^19^ According to the 2013 WHO-UNICEF report based on the Joint Reporting Form (used by every member country to report annually on immunization program indicators, such as coverage levels, wastage rates, and expenditures), there were more than 5,000 health districts (or similar subnational units) in WHO's Africa Region alone. A common problem is that authority and responsibility for immunization are divided in decentralized health systems. District-level managers are charged with optimizing immunization and other health services down to community levels. For immunization this includes maintaining herd immunity and controlling disease outbreaks. In low-resource settings, subnational decision-making processes are complex, mediated by both technical and political considerations and further challenged by constrained resources.^20^ Efforts at the national level to control funding for certain key services, including immunization, are sometimes misunderstood or viewed as contradictory to the very principles of decentralization.^21^^,^^22^

Some approaches to improving governance, organization, and management of routine immunization include:

- **Interagency coordinating committees:** In most LLMICs, a government-led ICC for immunization harmonizes planning and resource allocation to the programs from all major stakeholders. This forum provides a mechanism to improve coordination, collaboration, and cooperation among partners and the government, with varied success. In many LLMICs, external partners dominate the planning and resource allocation for routine immunization. Final decisions regarding input coordination, collaboration, and cooperation should be made by the host government.
- **Linkages:** Key technical and advisory bodies for routine immunization (NITAGs, NRAs, ICCs) need to be linked. Moreover, these institutions must coordinate their work with other disease control programs and analogous bodies at the health system level.
- **Pushing down to district levels:** Detailed microplans at the district level are recommended by WHO, outlining difficulties in accessing certain communities and subgroups. The plans cover delivery groups and strategies, how to reach such groups, including resource needs (equipment and staff) and means of monitoring these plans.

### Human Resources for Health: The People Factor

The growing complexity of immunization programs increases the need for a well-trained, capable health workforce. Technical skills must be adequate to administer an increasing number of vaccines to more children and expanded age groups. Management and supervisory skills become increasingly important as vaccine costs rise and financial and data management responsibilities increase. There are more opportunities for error, and the errors are costlier in terms of health risks to children and to the integrity of the entire immunization program. In many places, vaccinators represent the lowest tiers of health workers authorized to provide injections. Under such circumstances, task shifting of vaccine administration to community health workers or volunteers is not legal or appropriate. Despite the growing skill demands, the same basic approach to immunization training has been in use for more than 30 years. This usually takes the form of short, offsite, in-service training courses, often delivered through cascade training in which those providing the training may be experts in the subject matter but not in training techniques, or vice versa.

Immunization programs are growing in complexity, increasing the need for a well-trained health workforce.

Promising areas in which to invest to build the capacity and professional development of an appropriately trained health cadre include:

- **eHealth:** eHealth or mHealth technologies and processes have been shown to improve health worker capacity and effectiveness in other programs.^23^ With a global deficit of trained health workers^24^ and the rapid expansion of mobile phone networks in many LLMICs, the application of eHealth/mHealth to improve health communication and decision-making by health professionals (and clients) is increasingly recommended and feasible. E-learning opportunities can also support training needs and enhance learning.
- **Preservice training:** Some countries are working to improve immunization training, including preservice training. The aim is to build a foundation, at the outset of health workers' careers, which includes not just knowledge and skills but also professional values that directly affect routine immunization performance. These values include appreciating the importance of immunization data and understanding how to use them to improve management. Another value is to be respectful of the child caregivers such that they have a positive experience and return to complete the vaccination schedule.
- **Supervision:** Regular supervisory visits provide opportunities to reinforce good practices and values to improve services.

### Vaccine, Cold Chain, and Logistics Management

Cold chain and logistics management systems have been prominent features of the EPI blueprint from the onset. System requirements have expanded drastically over the past several years with the introduction of new vaccines and the frequent mass campaigns to control, eliminate, or eradicate specific diseases, such as polio, measles, rubella, and tetanus. While such initiatives tend to be well-resourced, the strain they place on the supply chain system are substantial and often under-recognized, particularly at subnational levels.^25^^,^^26^ The most visible impact of new vaccine introduction is an increase in the volume of products that need to be stored, transported, and tracked, as well as the need for more storage capacity due to the increased use of single-dose vials.

Introduction of new vaccines requires additional capacity of the supply chain system to handle the higher volume of products.

Successful cold chain and logistics management requires attention to many considerations. Adequate fuel and transport are necessary to ensure continuous running of cold chain equipment, which itself must meet international standards. Fuel and maintenance costs are often underestimated, and decisions to fund them are usually made by local governments, which may have other priorities. The resources that are allocated are sometimes diverted to other uses. Taken together, these circumstances lead to unreliable delivery of supplies and vaccine stock-outs. In addition, maintaining vaccines at proper temperatures has become more complex than in the past as some new vaccines are inactivated by exposure to freezing while other vaccines (those that have been in use for decades) are damaged by heat exposure. With the unit cost of newer vaccines far more expensive than those of the “original” complement of EPI vaccines, poor vaccine-handling practices have large financial consequences.

Managing the movement of vaccine products and supplies has largely been taken for granted over the decades, and a renewed interest in supporting this key element provides tangible opportunities to build efficient and effective systems to protect and handle the investments in vaccines.^25^ We are seeing new investments in:

- **Tools to support forecasting:** Tools for forecasting and monitoring of vaccines, supplies, and equipment stock help address a host of problems, from outdated inventory and inadequate storage space to inadequate stock and unmaintained equipment. Fortunately, new tools, such as improved supply chain and logistics management information systems, are becoming available.^27^ But they will only improve program performance if the required financing is in place and resources are correctly allocated and managed.
- **Vaccine technologies:** New developments are needed in this area to help countries meet increasing demands, including non-syringe delivery mechanisms and thermostable vaccines.^6^

### Quality and Reliable Service Delivery To Reach Every Child

**Figure 2.**
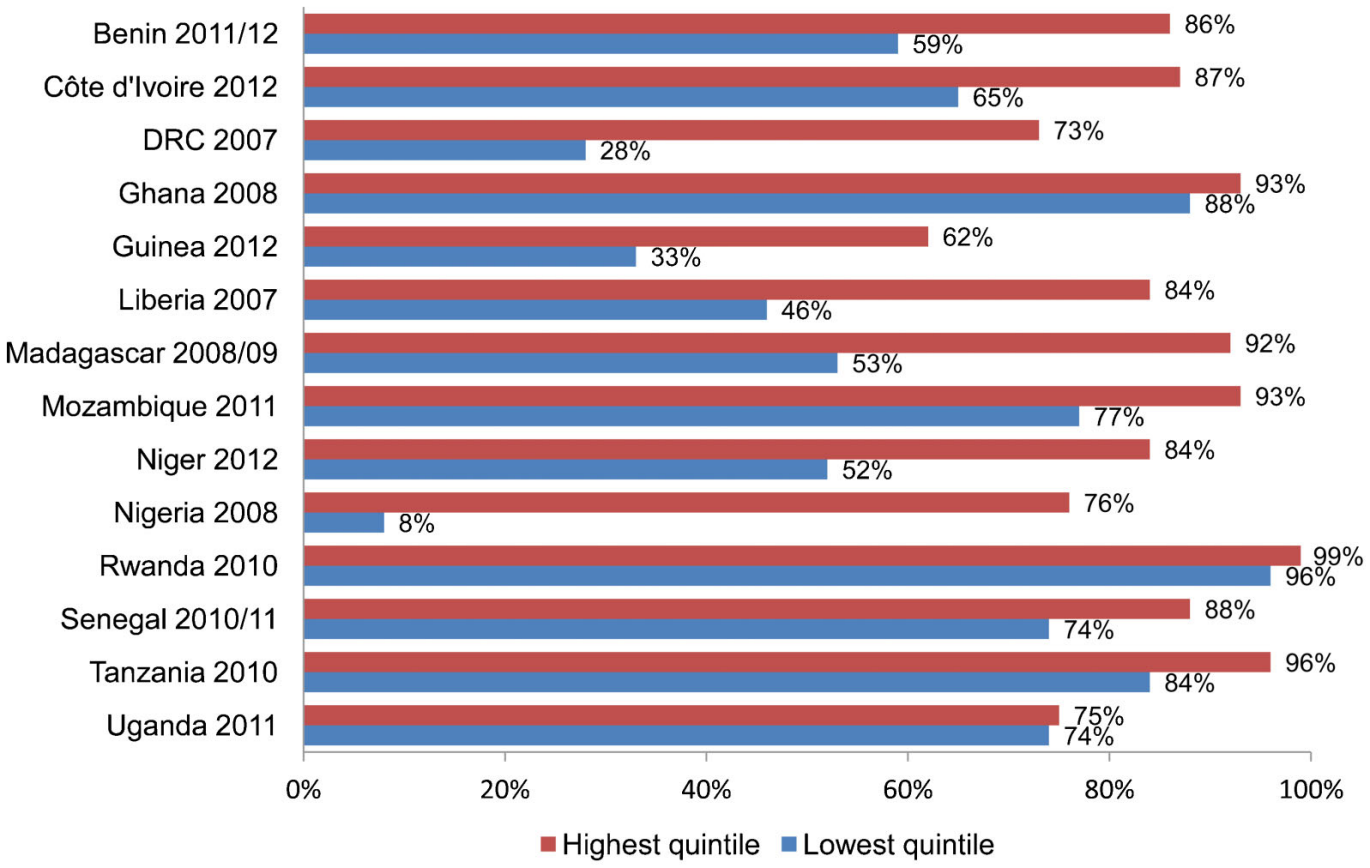
DTP3 Coverage for Lowest and Highest Wealth Quintiles in 14 African Countries, 2007–2012 Abbreviations: DRC, Democratic Republic of the Congo; DTP3, third dose of the diphtheria, tetanus, and pertussis vaccine. Source: Demographic and Health Surveys.^30^

Global coverage for the third dose of the diphtheria, tetanus, and pertussis vaccine (DTP3) increased from 73% in 2000 to 82% in 2008 but has remained stalled since then, hovering around 83%.^28^ Within-country variations are substantial, even in countries with high nationwide coverage.^29^ Data from the Demographic and Health Surveys from several African countries show that coverage is consistently lower in the poorest wealth quintile than in the highest quintile (Figure 2). The children who are missed tend to be those most in need of the protection that immunization confers.

Immunization coverage is consistently lower among the poor than among their wealthy counterparts.

Routine immunization typically employs two service delivery strategies, fixed and outreach. Fixed immunization takes place within health facilities while outreach entails visits to sites as far as 5–15 kilometers from the facilities. More resources are typically allocated for the former, leaving resource gaps for the latter.

Some approaches and strategies to improve the equity of services to efficiently and effectively deliver vaccines to every child include:

- **Finding the under-vaccinated (have not completed all recommended vaccinations) and unvaccinated (have not received any vaccinations):** Here again, microplanning—the detailed planning by individual health facilities and districts of how to reach all vaccine-eligible children in the catchment area—is essential for improving equity in immunization.^31^ Good planning requires knowledge of specific target populations, which is a serious challenge. As overall vaccine coverage levels rise, microplanning increasingly focuses on reaching hard-to-reach populations, whether defined in geographic, socioeconomic, cultural, or other terms.
- **Health worker–client interaction:** Not only must vaccinations be given properly and safely but the services must also be accessible and appealing enough for caregivers to bring their children for repeated visits. Under pressure to achieve low vaccine wastage rates for some vaccines such as measles, providers sometimes turn mothers away if they feel that not enough children have come to an immunization session to justify opening a multi-dose vial of vaccine. This has resulted in children being vaccinated too late or too early and contributes to high drop-out rates because of the added inconvenience it imposes on caregivers. Supporting health workers to balance the challenge of effectively and efficiently providing services is an important strategy to improving the delivery of services.

### Communication and Community Partnerships

Educating and mobilizing the public to support immunization and to use immunization services is central to EPI. In practice, this requires health workers and other trusted individuals to keep caregivers informed of where, when, and how many times they need to bring children for vaccination. Caregivers consistently cite health workers as their most important source of information on immunization, yet health workers receive limited training and supervision on interpersonal communication skills and its importance.

Caregivers consistently cite health workers as their most important source of information on immunization.

The acceptability of immunization is highly variable worldwide. For example, non-acceptance of immunization, or “vaccine hesitancy,” is a common problem in Europe,^32^ where vaccine-preventable diseases are not commonly encountered due to the success of immunization efforts. Particular groups disseminate misinformation, for example, linking the measles, mumps, and rubella (MMR) vaccine to autism.^33^ Challenges have ranged from isolated episodes of non-acceptance (due to religious, ethical, or medical considerations) to active mobilization against immunization programs driven by political or conspiratorial arguments.^34^

In LLMICs, demand for immunization is generally high and closely related to the availability of vaccines and quality of services,^6^ with non-acceptance limited to certain subpopulations. Revitalizing investments in communication to provide key information and direct the attention of caregivers and decision-makers to immunization is important.^34^^,^^35^ Non-use of services is sometimes attributed to mothers' distrust of immunization.^36^ Other reasons include perceived poor quality, unreliability, and inaccessibility of services.^3^^,^^37^^–^^39^ Persuading these populations to accept vaccines is complex. Knowing about vaccination, although important, does not lead to vaccine acceptance. Locally adapted and appropriate communication strategies are needed to address the sociocultural and political influences that impact immunization behavior.^34^

Waisbord and Larson (2005) identified 4 key challenges confronting immunization programs^34^:

1. Children do not get vaccinated if caregivers do not know the value of vaccines, when children need to be immunized, and where vaccines are administered.
2. Children do not get vaccinated when communities are excluded and beyond the reach of immunization services.
3. Children do not get vaccinated if caregivers do not trust the safety of vaccines.
4. Children do not get immunized when vaccines are not available.

Empirical results show that partnering with the community to develop communication strategies helps build trust and acceptance of vaccines and improve the quality, convenience, and use of vaccination services.^34^

Involving communities in immunization programs has proved to build trust and acceptance of vaccines.

The active participation of communities in planning and carrying out outreach services has been shown to be associated with vaccination coverage increases in 3 countries.^40^ In addition, in East Timor, India, and Nepal, community participation in monitoring immunization coverage within their own villages has contributed to service improvements. The involvement of nongovernmental organizations in maintaining a community scorecard on primary health care services was associated with an improvement in immunization coverage in Uganda.^41^ In Kenya, a process of community dialogue between community members and health personnel was so successful that it was added to the essential package of services.^20^^,^^42^

### Generation and Use of Quality Immunization Data

The generation of high-quality immunization data is important to informing programmatic decisions. The two main sources of vaccine coverage data are periodic population-based surveys, such as Demographic and Health Surveys (DHS) and Mixed Indicator Cluster Surveys, and routine administrative reports. Maintaining and improving the quality of routine immunization data is a constant challenge. Survey-based and administrative vaccine coverage figures for a given country or district are often discrepant. Routine administrative reports usually overestimate coverage relative to surveys. WHO/UNICEF coverage estimates from the Joint Reporting Form are often lower than official country-reported figures.^43^^,^^44^

One key problem is the inaccuracy of denominator (population) data, which are often based on outdated census data or inaccurate projections that do not reflect recent growth or population movement. Other common problems include double-counting of doses given, the mixing of doses given to older children with doses given to infants, and the fabrication of reports in order to achieve targets.^45^

Some approaches that have proved to be successful are:

- **Home-based immunization records such as vaccination or child health cards:** These are necessary for the accuracy of population-based surveys,^46^ and they serve as the cornerstone of vaccination programs at the local level. Health workers and caregivers are taught to value this document and that it should be requested and reviewed at every contact point between the caregiver and health worker. Yet home-based immunization records remain unavailable as they can often be out of stock and heavily underused. Of 23 DHS surveys conducted in Africa between 2010 and 2013, the median availability of cards was 68%, with only 28% and 29% of children having cards in Nigeria and Ethiopia, respectively.^47^^,^^48^
- **Better tools for data collection and analysis:** Better data can lead to better decisions and better health outcomes. For example, the use of vaccine registries can improve the accuracy in determining both the denominator for target populations and who and where the “unreached” are.
- **Expanded disease surveillance activities:** Frontline health workers routinely report cases of notifiable diseases, including vaccine-preventable diseases. In active surveillance systems, each facility must report the presence or absence of these cases, ideally on a weekly basis, more often realistically in many countries on a monthly basis. These raw data are fed into epidemiological surveillance systems, and the data are pooled and analyzed at higher levels.
- **Local use of programmatic data:** Routine immunization programs typically generate a wealth of data on programmatic indicators that can be used to improve program performance and services. Regular monthly and quarterly review meetings to examine such information at facility and district levels have been shown to improve routine immunization program performance.^40^^,^^49^ Data on cold chain functioning, frequency and place of immunization sessions, drop-out rates, and other indicators can be used by local health personnel to evaluate their own performance, identify gaps, and implement corrective actions to improve services.
- **Information feedback:** High-functioning immunization programs provide regular feedback reports to all levels of the health system, summarizing program outputs, cold chain performance, disease trends, and outbreak control activities. Feedback is essential to encourage the active participation of all health workers in the process of data collection and preservation of the integrity of the data for program and policy decision-making.

### Sustainable Vaccine and Immunization Financing

The benefits of vaccines extend beyond those accruing to the person being immunized. Immunization also reduces the potential transmission of bacteria and viruses to others not yet vaccinated. This herd immunity effect protects the unvaccinated, provided a threshold number of people are immunized and rendered immune. Eliminating epidemic childhood diseases reduces curative care costs^4^ and makes countries more attractive places in which to invest. These and other externalities—in particular, the herd immunity that immunization creates—qualify immunization as a public good, one that must be rationalized among other competing health interventions for government resources.

If enough people are vaccinated and rendered immune, immunization then also provides herd immunity, protecting the unvaccinated from disease.

Immunization, like other preventive services, sometimes falls victim to its own success, especially in low-resource settings. When coverage is continually high and outbreaks of vaccine-preventable diseases are averted (thereby reducing the vaccine-preventable disease burden), immunization demand may drop and policy makers and decision-makers may turn their attention and allocate resources to other interventions.^8^ The drop in resources for routine immunization usually results in a drop in coverage. Often the impact of these policies is not seen for a year or two. Immunity levels wane and vaccine-preventable disease outbreaks return, incurring higher containment costs and political threats to governments.

**Figure 3.**
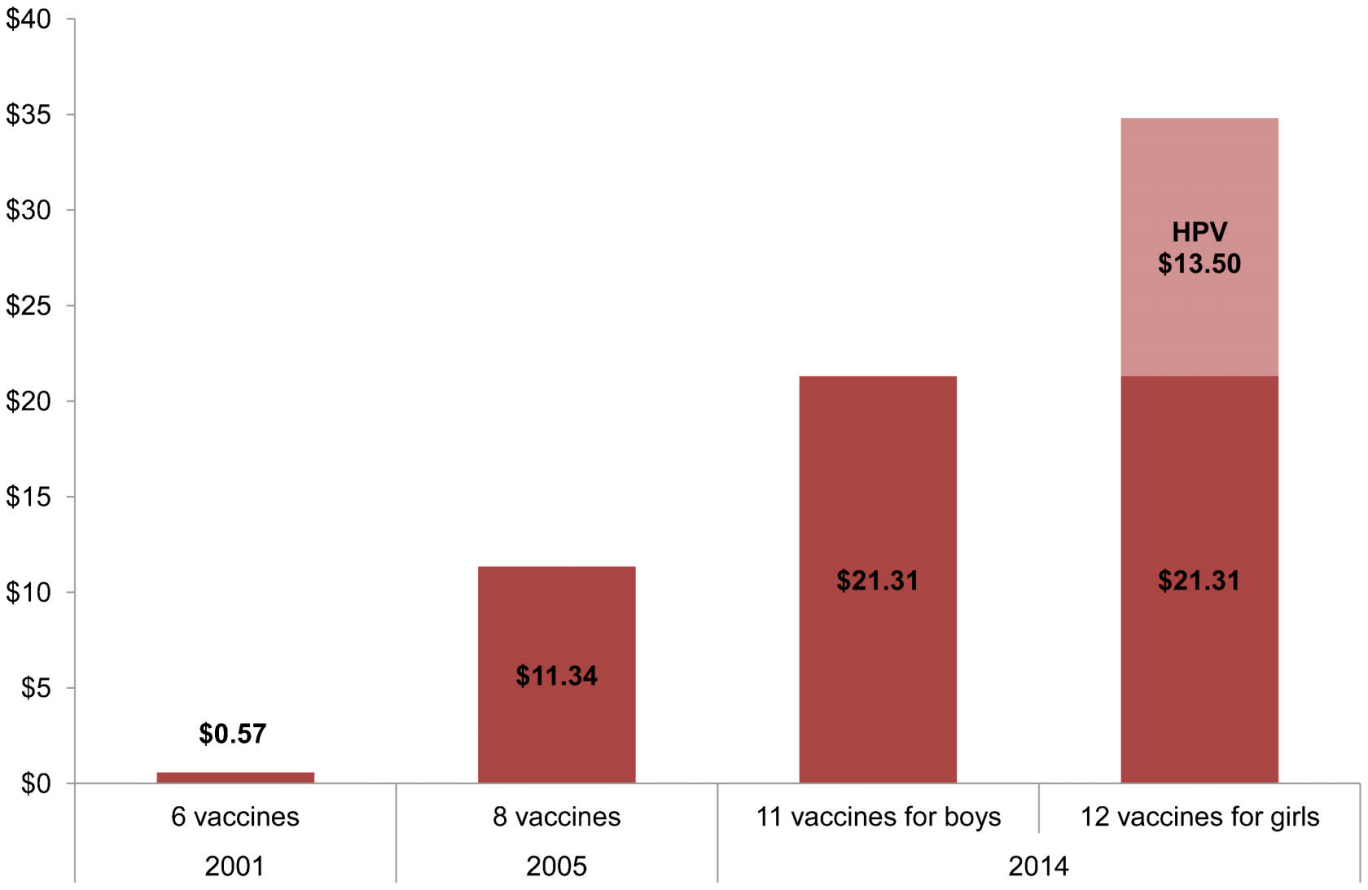
Vaccine Costs per Child (US$) for Routinely Recommended Vaccines^a^ From Birth Through Age 18, 2001, 2005, 2014 Abbreviation: HPV, human papilloma virus. Source: UNICEF contract prices as of May 8, 2014.^7^ Costs based on lowest-available price to UNICEF. ^a^ The 2001 WHO-recommended vaccination schedule comprised 4 vaccines containing 6 antigens: diphtheria, tetanus, pertussis (DTP); measles; polio; and bacille Calmette-Guérin (BCG). In 2005, 2 more antigens were added: hepatitis B (Hep B) and *Haemophilus influenzae* type b (Hib). In 2014, rotavirus, pneumococcal conjugate, rubella, and HPV were added to the schedule, bringing the total number of WHO-recommended antigens to 12. Notes: The WHO-recommended target group for HPV vaccination is girls ages 9–13 years. HPV vaccination of boys is optional but not recommended in resource-constrained settings. WHO recommends all countries introduce at least 1 dose of inactivated polio virus (IPV) into their immunization schedules by the end of 2015, which will add an additional $1.26 to the schedule.

In many LLMICs, immunization budgets are currently insufficient to sustain programs and incorporate the new, costlier vaccines, although there is fiscal space to absorb costs.^50^ New vaccines have added both tremendous benefit and costs to the original EPI blueprint.^7^ For example, the Gavi/UNICEF cost of the human papillomavirus (HPV) vaccine to protect girls against cervical cancer is about US$4.50 per dose, with 3 doses recommended (total cost $13.50). In 2001, the total cost of the original set of 6 WHO-recommended vaccines was under US$1 (Figure 3). With the introduction of a combination 5-in-1 pentavalent vaccine in 2005, children became protected against 2 additional vaccine-preventable diseases, and the total vaccine costs increased to about $11. In 2014, the total cost of 11 WHO-recommended vaccines reached approximately $21, with an additional $13.50 to vaccinate girls against HPV. Looking forward, adding an inactivated polio vaccine (IPV) to support the polio eradication efforts will add $1.26 to the schedule, bringing the total vaccine costs to about $23 for boys through age 18 and to $36 for girls. Note that prices vary by brand of vaccine and vaccine presentation (eg, multi-dose vials, liquid formulation). Delivery costs, estimated by Lydon and colleagues (2014), add about $25 per child, bringing the total delivery costs to fully immunize a child to around $50 to $60.^7^^,^^51^ These delivery costs are most likely underestimates.

It could cost up to an estimated US$60 to vaccinate 1 child when taking into account both delivery and vaccine costs.

**Figure 4.**
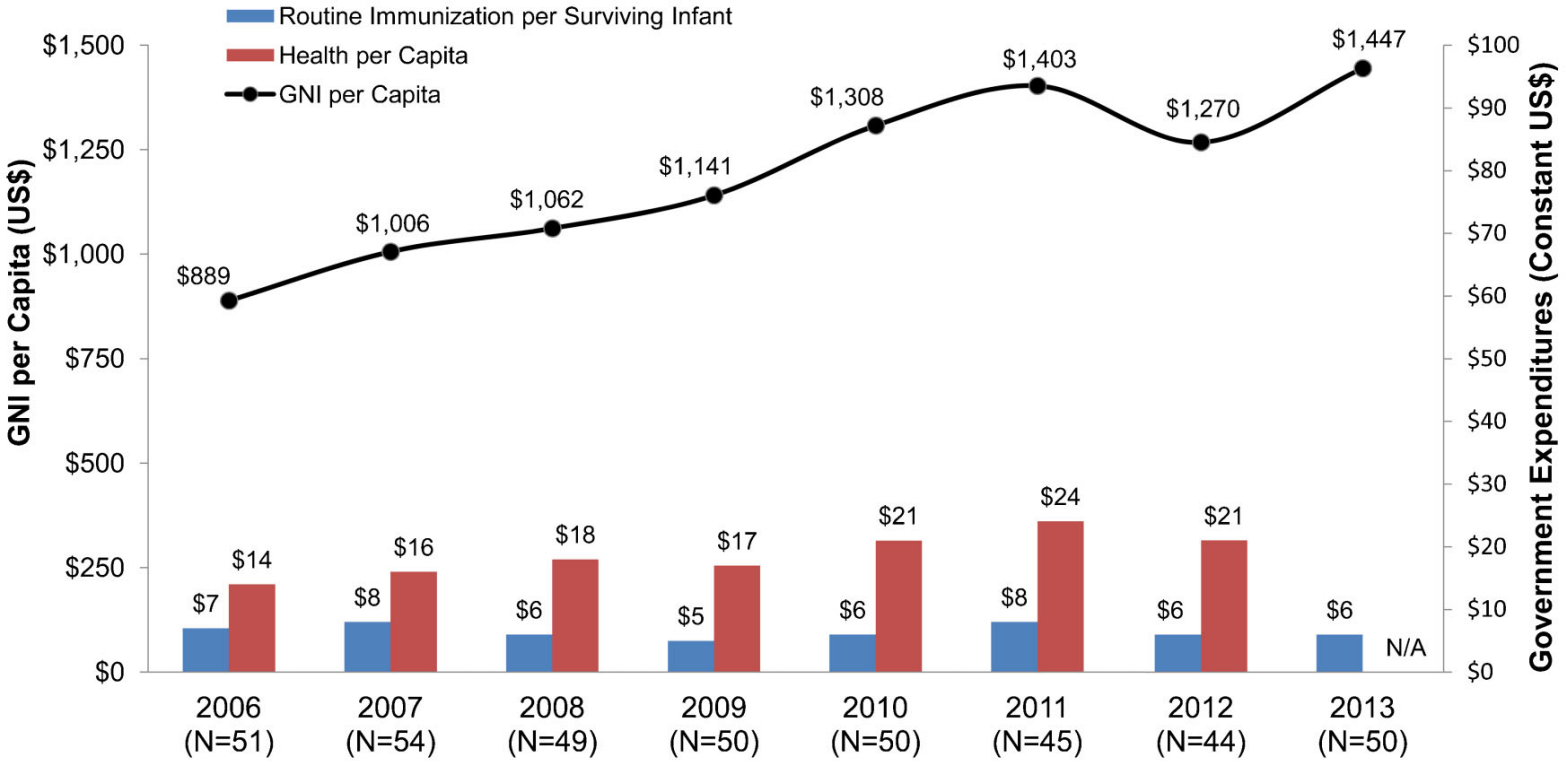
GNI per Capita and Government Expenditures on Health per Capita and on Routine Immunization per Surviving Infant Among Reporting Gavi-Eligible Countries Abbreviations: GNI, Gross National Income; N/A, not available. Source: Routine immunization expenditures extracted from the WHO/UNICEF Joint Reporting Form, Immunization Financing Database,^53^ Indicator 6500. Expenditures reported in local currency were converted to US$ using the midyear exchange rate. Surviving infant populations derived from the UN Population Division Online Database.^54^ GNI per capita (Atlas method) expressed in US$ and extracted from the World Bank.^55^ Government expenditures on health extracted from the WHO National Health Account Database.^56^ All values in population-weighted constant (2013) US$. The following country-years were excluded from the analysis: Bhutan 2007, Uganda 2011, and Uzbekistan 2006, 2009.

Some current approaches toward sustainably financing immunization include:

- **Establishing legal frameworks:** Increasing national immunization budgets and protecting those budgets through legislation helps countries achieve country ownership. Current work toward establishing legal frameworks for immunization financing provides a foundation for sustainably financing immunization. For example, Nigeria, Africa's most populous country, more than doubled its immunization budget from 2010 to 2012 and passed a new national health bill that includes a provision explicitly guaranteeing public immunization financing.^52^
- **Tracking immunization program expenditures:** Immunization program expenditures are notoriously hard to track. Improved resource-tracking down to district levels provides the basis to improve budget and resource allocation. Between 2006 and 2013, government spending on routine immunization per surviving infant remained relatively flat while health per capita and gross national income generally rose, data from Gavi eligible countries show (Figure 4). To compound this picture, the cost of newer vaccines has been rising, from about US$0.57 per child in 2001 (for 6 antigens) to approximately $21 per boy and $35 per girl in 2014 (for 11 antigens), as mentioned earlier (Figure 3). The current nominal cost of the vaccines alone to vaccinate 1 child is equivalent to or exceeds the amounts many LLMICs have spent on all their public health programs combined, which has ranged, on average, from $21 to $24 per capita since 2010 (Figure 4).

## GAVI AND GRADUATION

Financing vaccine purchase and immunization delivery programs is increasingly challenging, particularly to LLMICs unaccustomed to such high health spending. Gavi, an innovative financing mechanism created in 2000 to accelerate access to new and underutilized vaccines, prepays production costs for the newer WHO-recommended vaccines directly to manufacturers and provides grants to the 73 poorest LLMICs to finance procurement and introduction of the vaccines into the routine system. The vaccines are procured through UNICEF's Supply Division, which, independently of Gavi, is the world's largest vaccine procurer. About half of UNICEF-procured vaccines currently go to countries not supported by Gavi.^57^ Another example of a collective vaccine procurement mechanism is the Pan American Health Organization's Revolving Fund, a collective bargaining mechanism of pooled procurement for the WHO region of the Americas.^58^

In the 73 poorest LLMICs, Gavi finances nearly all the newer vaccines. By 2015, the first 20 countries will begin graduating from Gavi eligibility as their per capita gross national incomes already exceed the Gavi funding threshold (US$1,500 in 2010, adjusted in 2014 to $1,570). Upon graduation, the countries will need to self-finance all their vaccines.^17^ Although Gavi facilities this transition, countries will face global market vaccine prices, joining some 40 middle-income countries that were never eligible for Gavi support. The uncertainty of future vaccine prices is an impediment to planners and legislators, particularly as they write new or revise existing vaccine legislation obligating their governments to finance the programs. The domestic investment case in countries is often not properly developed. That said, countries are graduating from Gavi assistance at a time when they are experiencing robust economic growth. Assuming continued economic growth, countries, with political support and commitment, could fully finance their own programs.^59^

Prerequisites for sustainable financing include:

- **Data:** Cost-effectiveness studies are needed for each vaccine to inform decisions to introduce into national immunization schedules.^51^ Few LLMICs have the necessary research expertise to carry them out.
- **Effective national procurement and regulatory authorities:** National procurement and regulatory bodies have a role to ensure that governments purchase safe, effective vaccine products at the lowest possible cost.^17^ Investments in the ability of countries to manage procurement and supply are important to countries nearing graduation.^17^ This is largely because, depending on the source of vaccine (ie, procurement through a United Nations Agency, self-procurement, or domestic vaccine producer), countries need to strengthen the management of procurement including product pricing strategies. National regulatory functions vary depending on procurement mechanisms. At a minimum, countries must have a regulatory system for market authorization, licensing activities, and pharmacovigilance in order to procure vaccines for use in immunization programs. This function is separate from vaccine procurement duties. (If countries self-procure vaccines, their regulatory authority must be able to perform two additional functions—lot release and laboratory access. If a country produces vaccines domestically, they must also perform regulatory inspections and regulatory oversight of clinical trials.)
- **Public finance systems:** Performance of public finance systems must be adequate to efficiently handle the increased immunization program budgets, and managers must be adept enough to demonstrate value for money.^60^

## WHERE DO WE GO FROM HERE?

Ultimately, any vaccine, whether part of the original EPI blueprint or new, is only as effective as the health system that delivers it. To reach every child, it will be essential to address inequities in access to vaccines (often masked by high national coverage), strengthen the fragility of underlying health systems, and invest directly in routine immunization. Countries have pledged to achieve ownership of their immunization programs by finding sustainable financing solutions and developing needed institutional innovations. If the resulting country and external partner investments materialize, immunization services will reach all children, continuously, using locally appropriate strategies.^61^

However, EPI's history shows that without continued and sustained investments in long-term development approaches, gains can be lost.^2^^,^^62^ With the push to universal immunization in the 1980s, the global solidarity to accelerate coverage resulted in unprecedented increases in coverage for the basic set of EPI vaccines by the end of 1990. However, the focus on increasing coverage, as opposed to building sustainable and equitable health care systems, was evident as coverage stagnated throughout the 1990s, with global and regional averages masking lower local coverage, particularly in the African region where coverage levels for fully immunized children are under 50%.^62^ The reason for this is not only weaknesses in the health system and lack of continued investment in routine immunization but also the underlying funding pressures that divert resources away from routine immunization. Investments to strengthen routine immunization systems have been limited, instead mediated through broader bilateral or multilateral health system strengthening strategies or as a side benefit of specific disease control initiatives.^62^

Countries have made undeniable progress and significant advances in routine immunization over the last 40 years. It is costing more to save a life from vaccine-preventable diseases than before, but that is because there are more lifesaving vaccines now and immunization is saving more lives than ever before. We are at a time when new and future vaccines will be on the order of dollars versus cents, and recurrent costs to support delivery and administration of vaccines must be factored into the budget equation, whereas it had not been in the past. Over the past 4 decades, progress has clearly been made in building the 8 critical components of routine immunization. Expanding these lessons to support the primary health system provides opportunities to address the constant barriers that choke the primary health care system. Transformative changes are required to support and manage a trained and skilled workforce, shore up infrastructure weaknesses, and improve data and information needs. These areas are not new cries for attention.^14^ Addressing these underlying deficiencies that underpin primary health care systems are investments in the future dividend of a nation's health and productivity.

## Notes

**Peer Reviewed**
